# Cytotoxicity Induced by Newly Synthesized Palladium (II) Complexes Lead to the Death of MCF-7 and MDA-MB-435 Cancer Cell Lines

**DOI:** 10.34172/apb.2023.017

**Published:** 2021-10-10

**Authors:** Bruna Alexandre Oliveira da Silva, Isabela Spido Dias, Luís Eduardo Sarto, Elba Pereira de Gois, Claudia Torres, Eduardo Tonon de Almeida, Cibele Marli Cação Paiva Gouvêa

**Affiliations:** ^1^Institute of Natural Sciences, Federal University of Alfenas, Alfenas, Brazil.; ^2^Federal Institute of Education, Science and Technology of the South of Minas Gerais, Machado, Brazil.; ^3^Institute of Chemistry, Federal University of Alfenas, Alfenas, Brazil.

**Keywords:** Anticancer agent, Breast cancer, Cell migration, Cell viability, Melanoma, Metal complexes

## Abstract

***Purpose:*** Breast cancer is the most common female malignancy and melanoma is the most lethal type of skin cancer. Traditional therapy for cancer treatment is far from satisfactory due to drug resistance and side effects, thus a search for new medicines is being emphasized. Palladium(II) complexes have been reported as anticancer potential agents. In this work, the anticancer activities and cell death induction of a new series of square-planar Pd(II) complexes were evaluated against MCF-7 and MDA-MB-435 cancer cells.

***Methods:*** MCF-7 (breast carcinoma) and MDA-MB-435 (melanoma) cells were cultivated, and treated with ligand and Pd(II) complexes. Cell growth, migration and adhesion inhibition, morphological alterations, cell death induction and, DNA interaction upon treatment were studied.

***Results:*** Pd(II) complexes exhibited both short and long-term antiproliferative effects on both cell lines, reducing by 80% cell growth in the SRB assay and abolishing longterm proliferation, estimated by the clonogenic assay. Complexes reduced significantly (*P*<0.05) cell migration and adhesion when compared to the control group. Complexes induced morphological alterations in cell lines and significant (*P*<0.05) cellular shrinkage. Cell death was induced and the complexes were able to interact with DNA, inducing cleavage of double-stranded DNA, which may account for the complexes cytotoxic effects, observed against both MCF-7 and MDA-MB-435 cells.

***Conclusion:*** Overall, the complexes exhibited cytotoxic activities and induced cell death. These observations emphasize an anticancer role with a potential therapeutic value for Pd(II) complexes to improve the outcome of patients with breast cancer and melanoma.

## Introduction

 Cancer is the second leading cause of mortality worldwide, with increasing rates of incidence and disability. Breast cancer is the most common diagnosed malignancy, being skin cancer the third most common, and they have been on the rise for several years.^1-3^

 Breast cancer is a highly curable disease when detected early. Nevertheless, patients usually respond differently to therapies and present diverse outcomes, demonstrating the extensive diversity of the various cancer subtypes. The differential responses to treatment may lead to a rapid cancer progression, an increased degree of malignancy and, a high mortality rate.^4,5^ Once a metastatic disease is established, the response to the same treatment strategy becomes dismal. Despite recent advances in treatment, metastatic and advanced breast cancer will be incurable, being cytotoxic chemotherapy the only treatment option.^4-6^

 Melanoma is the most aggressive and deadly form of all skin cancers with a poor prognosis once the disease enters metastasis. The incidence rate of cutaneous melanoma is increasing rapidly in both men and women.^7,8^ The approval of tyrosine kinase and immune checkpoint inhibitors have been revolutionized the treatment of cutaneous melanoma.^7,9,10^ The efficacy of currently available treatment schemes for advanced melanomas is low, expensive, and hampered by significant side-effects and resistance development to targeted therapies, remaining chemotherapy an important treatment option.^11-14^

 Cisplatin, the square planar Pt(II) complex, cis-[PtCl_2_(NH_3_)_2_], has played a chemotherapeutic key role among the metal-based anticancer agents.^15^ However, the occurrence of cisplatin side effects and drug resistance has stimulated the interest in the design of new complexes with better antitumor activities and lower toxicity than platin agents.^16,17^ The similarity between the coordination chemistry of platinum(II) and palladium(II) leads to the exploration of palladium(II) complexes as alternative therapies for the treatment of cancer.^17^

 In pursuing our interest in the metal complexes and biological activity of Pd(II) compounds,^18-21^ we report herein the synthesis, characterization and, cytotoxic effects of new Pd(II) complexes on MCF-7 and MDA-MB-435 cell lines.

## Materials and Methods

###  Synthesis of imine ligand (L) and Pd(II) complexes (C1-C6)

**Figure 1:**
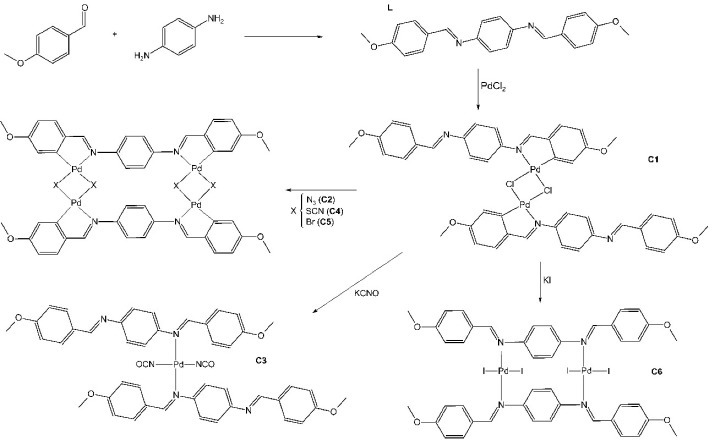
Schema for the synthesis of ligand (L) and Pd(II) complexes (C1-C6).

 The imine ligand (Schiff Base), H_2_ani_2_*p*-pheny (**L**), was prepared by slowly adding a solution of *p*-phenylenediamine (16.0 mmol,1.7773 g, Acros, USA) dissolved in 5.0 mL ethanol (Ecibra, Brazil) to a solution of *p*-anisaldehyde (32.0 mmol, 4.4817 g, Carlo Erba, Spain)dissolved in 2.0 mL ethanol for 3h, at room temperature. The starting Pd(II) complex C1 was prepared according to the previously described method.^22^ Briefly, the complex C1 was synthesized by adding L (5.64 mmol, 1.9477g) to a solution of PdCl_2_ (5.64 mmol, 1.0000 g, Sigma, USA) dissolved in 50 mL methanol (Impex, Brazil)). The resulting solution was stirred at room temperature for 8 hours and an orange pellet was formed. C1 was the starting complex to prepare C2-C6, by the substitution of Cl by other halides and pseudohalides. The other complexes were prepared by adding NaN_3 _(0.618 mmol, 0.0456 g, Riedel, USA), KCNO (0.618 mmol, 0.0543 g, Carlo Erba, Spain), KSCN (0.618 mmol, 0.0658 g, Merck, Germany), KBr (0.618 mmol, 0.0734 g, Vetec, Brazil) or KI (0.618 mmol, 0.1023 g, Merck, Germany) to C1 (0.309 mmol, 0.3082 g) dissolved in 20.0 mL acetone to yield **C2**, C3, C4, C5 and C6 complexes respectively. The resulting solutions were stirred at room temperature for 2 hours and a pellet was formed which was filtered off, washed (with water and ethyl ether) and vacuum-dried. The schemata for the synthesis of L and C1-C6 are given in Figure 1.

###  Characterization 

 Characterization of the compounds was carried out as described elsewhere.^23,24^ Melting or decomposition points were determined on Marte (PFM II; Brazil) equipment. Elemental Analysis (C, H, N) were determined using a Leco Instruments Elemental Analyzer TruSpec, model CHNS-O (USA). The IR spectra were obtained using a Shimadzu IR Prestige-21 (Japan), KBr pellets technique and, 4000-400 cm ^-1^ for spectral range. The ^1^H NMR spectra were obtained by using a Bruker AC-200 (USA) spectrometer at 300 MHz and DMSO-d_6_ was used for sample preparation. Thermal analyses (TG-DTA) were carried out using a TA Instruments (USA) thermobalance SDT Q 600, underflow of dry synthetic air (100 mL/min), temperature up to 1100°C and heating rate of 20°C.min^–1^, in α-alumina sample holders, using sample masses of about 10.0 mg. The patterns of powder X-ray diffraction were obtained by Rigaku Ultima IX equipment (Japan) using K_α_Cu wavelength (k = 1.5418 Å) setting of 34 kV and 20 mA. The molar conductivity measurements were made in CH_3_NO_2 _solution (10^−4^ M) using an AAker equipment. The cell fitted with a platinum electrode was calibrated with 10 ^-3^ M aqueous KCl solution. The cell constant was 1.0 cm ^-1^, and KCl molar conductivity was found to be 1.413 μS/cm, at 25°C. The solutions of prepared complexes were studied in a concentration of 10 ^-4^ M, at 25°C.

 L yellow solid, M.p.: 213°C; Anal. Calc. (%) for C_22_H_20_N_2_O_2 _(344.40 g/mol): C, 76.72; H, 5.85; N, 8.13; Found (%): C, 76.54; H, 5.31; N, 8.04.; IR (KBr pellet, cm ^-1^): 2970, 2873, 1604, 1022, 844; ^1^H NMR *δ* ppm (DMSO-*d6*, 300 MHz): 8.58 (s, 1 H), 7.89 (d, 2H, *J*=8.92 Hz), 7.06 (d, 2H, *J*=8.69 Hz), 3.83 (s, 3H); molar conductivity: 4.63 μS/cm.

 C1 orange solid, M.d.: 258°C; Anal. Calc. (%) for [Pd(µ-Cl)(C_22_H_19_N_2_O_2_)]_2_ (970.54 g/mol): C, 54.45; H, 3.95; N, 5.77; Pd 21.57; Found (%): C, 53.75; H, 4.51; N, 6.90; Pd 21.83 (TG); IR (KBr pellet, cm ^-1^): 2933, 2904, 1595, 1018, 835; ^1^H NMR *δ* ppm (DMSO-*d6*, 300 MHz): 8.58 (s, 1H), 7.85 (d, 2H, *J*= 8.92Hz), 7.12 (d, 2H, *J*=8.69 Hz), 3.85 (s, 3H); molar conductivity: 6.89 μS/cm.

 C2 orange solid, M.d.: 149°C; Anal. Calc. (%) for Pd_4_(N_3_)_4_(C_22_H_18_N_2_O_2_)_2_ (1278.54 g/mol): C, 41.76; H, 2.95; N, 16.60; Found (%): C, 41.76; H, 3.15; N, 17.45; IR (KBr pellet, cm ^-1^): 2933, 2837, 2063, 1600, 1024, 833; ^1^H NMR *δ* ppm (DMSO-*d6*, 300 MHz): 8.52 (s, 1H), 7.87 (m), 7.02 (d, 2H, *J=*8.69 Hz), 6.33 (m), 3.83 (s, 3H); molar conductivity: 9.26 μS/cm.

 C3 orange solid, M.d.: 150°C; Anal. Calc. (%) for Pd(NCO)_2_(C_22_H_20_N_2_O_2_)_2_ (879.21 g/mol): C, 62.54; H, 4.59; N, 9.46; Pd 11.86; Found (%): C, 62.29; H, 3.96; N, 8.46; Pd 10.09 (TG); IR (KBr pellet, cm ^-1^): 2929, 2839, 2177, 1595, 1026, 835; ^1^H NMR *δ* ppm (DMSO-*d6*, 300 MHz): 8.58 (s, 1H), 7.89 (d, 2H, *J*=8.69 Hz), 7.06 (d, 2H, *J*=8.69 Hz), 3.83 (s, 3H); molar conductivity: 8.10 μS/cm.

 C4 violet solid, M.d.: 145°C; Anal. Calc. (%) for Pd_4_(*µ*-SCN)_4_(C_22_H_18_N_2_O_2_)_2_ (1342.79 g/mol): C, 42.93; H, 2.70; N, 8.34; Pd 31.70; Found (%): C, 41.50; H, 3.91; N, 10.50; Pd 30.86 (TG); IR (KBr pellet, cm ^-1^): 2931, 2833, 2110, 1595, 1028, 827; ^1^H NMR *δ* ppm (DMSO-*d6*, 300 MHz): 8.48 (s, 1H), 7.79 (m), 7.02 (d, 2H, *J*=8.69 Hz), 6.40-6.60 (m), 3.80 (s, 3H); molar conductivity: 8.67 μS/cm.

 C5 orange solid, M.d.: 204°C; Anal. Calc. (%) for Pd_4_(*µ*-Br)_4_(C_22_H_18_N_2_O_2_)_2_ (1430.07 g/mol): C, 36.95; H, 2.54; N, 3.92; Pd 26.06; Found (%): C, 35.94; H, 3.31; N, 4.35; Pd 27.76 (TG); IR (KBr pellet, cm ^-1^): 2926, 2852, 1591, 1020, 835; ^1^H NMR *δ* ppm (DMSO-*d6*, 300 MHz): 8.58 (s, 1H), 7.85 (m), 7.02 (d, 2H, *J*=8.69 Hz), 6.50-6.58 (m), 3.80 (s, 3H); molar conductivity: 10.24 μS/cm.

 C6 brown solid, M.d.: 190°C; Anal. Calc. (%) for Pd_2_I_4_(C_22_H_19_N_2_O_2_)_2_ (1407.24 g/mol): C, 37.50; H, 2.86; N, 3.98; Pd 14.72; Found (%): C, 36.95; H, 3.36; N, 4.40; Pd 15.19 (TG); IR (KBr pellet, cm ^-1^): 2924, 2831, 1591, 1022, 831; ^1^H NMR *δ* ppm (DMSO-*d6*, 300 MHz): 8.58 (s, 1H), 7.85 (d, 2H, *J*=8.69 Hz), 7.02 (d, 2H, *J*=8.69 Hz), 3.80 (s, 3H); molar conductivity: 16.75 μS/cm.

###  Cell culture and treatment

 The MCF-7 and MDA-MB-435 cell lines were purchased from the Rio de Janeiro Cell Bank (Brazil). Cells were cultured in RPMI 1640 (Cultilab, Brazil), supplemented with 20% (v/v) fetal bovine serum (Cultilab, Brazil), penicillin and streptomycin (Cultilab, Brazil), and maintained in a humidified atmosphere at 37°C, with 5% CO_2_. The medium was replaced every two days and cells were sub-cultured every 3 days, after 0.25% Trypsin-EDTA solution treatment.^18^ Exponentially growing cells viability was assessed by the Trypan blue (Sigma, USA) dye exclusion method before each experiment whereupon the cells were plated and left overnight to allow for attachment. The cells were then exposed to L and C1-C6 different concentrations. Compounds were freshly dissolved in dimethyl sulfoxide (DMSO, Sigma, USA), sterilized by filtration and, diluted in the culture medium. Treated-cells with vehicle were the negative control (NC), and with cisplatin, the positive (PC).

###  Sulforhodamine B proliferation assay

 The short-term antiproliferative effect of the compounds was evaluated by the sulforhodamine B (SRB, Sigma, USA) assay, as described elsewhere,^25^ with some modifications.^18^ Briefly, 2×10^4^ cells/mL were seeded onto 96-well plates and, treated with different concentrations of compounds (0.05-5.0 µg/mL) for 24 hours. After treatment, cells were fixed with 10% trichloroacetic acid (w/v), washed and, dried for 24 hours. Cells were then stained with 0.4% SRB dissolved in 1% acetic acid (v/v), washed with 1% acetic acid and, allowed to dry. The well content was dissolved by adding 100 µL of 10 µM Tris, pH 10.5 and the absorbance of each well was measured at 510 nm using a Biochrom Asys UVM (USA) 340 microplate reader. The results obtained were used for GI_50_ (growth inhibition 50%, the concentration necessary to inhibit the cell growth by 50%) determinations, calculated relative to the negative control.

###  Clonogenic assay

 Long-term survival and proliferation after treatment were evaluated using a clonogenic assay. Exponentially growing cells were seeded (100 cells/well) into 24-well plates, treated with 0.05, 1.0 and, 5.0 µg/mL compounds for 24 hours, then the medium was removed and a fresh medium was added to the cells. Surviving cells were left to form colonies for 14 days, with medium replacement every 2 days. Henceforward, cells were fixed and stained for 30 minutes, with 6% glutaraldehyde (Exodo Cientifica, Brazil), containing 0.5% crystal violet (Neon, Brazil). Colonies (containing more than 50 cells) were counted under a light microscope and the survival rate of each group was calculated according to the corresponding plating efficiency.^26^

###  Wound healing assay

 The confluent monolayer of cells was scratched with a tip, and each well was rinsed with phosphate-buffered saline (PBS) to remove non-adherent cells. The cells were treated with 0.05 µg/mL compounds then incubated for up to 48 hours. Cell migration was observed under a microscope (Olympus BX52, Japan) at 24 hours intervals. The percentage of wound closure was calculated comparing the initial distance between both sides of the scratch with the distance between both sides of the scratch at the measured time.^27^

###  Extracellular matrix adhesion assay

 Before seeding cells (1×10^4^ cells/well), 96-well plates were coated with the extracellular matrix gel from Engelbreth-Holm-Swarm murine sarcoma (50 µL, Sigma, USA) and blocked by 1% bovine serum albumin (Sigma, USA) solution, for 1 hour at 37^o^C, whereupon cells were treated for 1 hour, with 0.05 µg/mL compounds. After treatment, non-attached cells were removed with gentle washing, and attached cells were fixed and stained for 30 minutes, with 6% glutaraldehyde, containing 0.25% crystal violet. The percentage of attached cells was calculated relative to the negative control, by counting cells under a light microscope (Olympus BX52, Japan).^28^

###  Morphological and morphometrics analysis

 Cells (2×10^4^ cells/mL) were cultured on coverslips, treated for 24 hours with 0.05 µg/mL compounds, fixed with 70% acetone for 15 min, washed with PBS, and stained with hematoxylin-eosin (Sigma, USA). Slides were mounted in Entellan (Merck, USA) and observed using an Olympus BX52 (Japan) microscope and Motic Images Plus 2.0 software (China). Fifteen random fields were analyzed per treatment, and cell digital images were acquired to describe cell morphology. Cell length was determined by measuring 150 cells per treatment.^18,21^

###  Cell death assay 

 Cell death was estimated using the Fast green-dye exclusion method,^29^ with modifications.^30^ Cells (2×10^4^ cells/mL) were cultured on coverslips, treated for 24 h with 0.05 µg/mL compounds, stained with 2% Fast green (Sigma, USA), followed by hematoxylin-Floxin (Sigma, USA), and slides were mounted in Entellan (Sigma, USA). Based on this method, the reddish-pink stained cells are viable, as they exclude Fast green, while green-stained cells are dead, as they are unable to exclude Fast green. We analyzed 600 cells/treatment using an Olympus BX52 (Japan) microscope and Motic Images Plus 2.0 software (China), to obtain the percentage of dead cells.

###  DNA cleavage study

 DNA cleavage activity of Pd(II) complexes was studied using DNA ladder pattern formation and plasmid DNA cleavage assays. Cells were incubated for 48 h with 0.05 µg/mL compounds and cell genomic DNA was extracted using the DNeasy Blood and Tissue Kit (Qiagen, USA) according to the manufacturer´s procedure. The DNA ladder, an indicator of apoptosis, was visualized by electrophoresis. Plasmid DNA cleavage studies were performed using 500 ng pUC19 DNA (Invitrogen, Lithuania) treated for 30 minutes with 0.05 µg/mL compounds. After incubation, 5 µL of each sample was separated by electrophoresis. Electrophoresis was carried out in a 1% agarose gel, at 80 V. Gel was stained with ethidium bromide (Invitrogen, Scotland) and the DNA bands were visualized by a UV light transilluminator. Plasmid DNA cleavage was analyzed by the conversion of supercoiled (SC) to open circular (OC) DNA form.

###  Statistical analysis

 Obtained data were compared by one-way analysis of variance (ANOVA) using GraphPad Prism software (version 6.0), followed by Tukey’s test when the *P *value was less than 0.05. Data are shown as the mean ± standard error of the mean (SEM) of three independent experiments.

## Results and Discussion

###  Synthesis and characterization

 The results of elemental analyses and spectral studies provided support for the suggested structures of the compounds (Figure 1). C1 presented square planar palladium chelated to nitrogen and carbon. The substitution of C1-Cl by other halides and pseudohalides produced complexes with two square planar chelated (C2, C4 and, C5). The replacement of a Cl- by NCO- and I- caused C1 deformation, forming Pd(II) complex with one and two square planar coordination with iminic nitrogen (C3 and C6 complexes, respectively).

###  Antiproliferative effect

 Pd(II) complexes exhibited both short and long-term antiproliferative effects. Cell proliferation decreased upon compound treatment (Table 1), being C6 the most active Pd(II) complex to both cell lines and the ligand the lowest active compound. Pd(II) complexes were more active to MDA-MB-435 than to MCF-7 cells (*P*<0.01), and more active than cisplatin.

**Table 1:** Short term growth inhibition of MCF-7 and MDA-MB-435 cancer cell lines upon treatment

**Compound**	**MCF-7**		**MDA-MB-435**	
	**GI**_max_ ** (%) **	**GI**_50_ ** (µg/mL)**	**GI**_max_ ** (%) **	**GI**_50 _ **(µg/mL)**
Cisplatin	62.21±1.38^a^	0.0544±0.0004^a^	76.06±0.22^a^	0.0384±0.0004^a^
L	57.75±1.87^b^	0.0660±0.0004^b^	70.47±1.39^b^	0.0521±0.0004^b^
C1	74.21±1.04^c^	0.0430±0.0003^c^	82.55±0.77^c^	0.0331±0.0001^c,e^
C2	69.48±1.38^d^	0.0488±0.0003^d^	78.85±0.40^a^	0.0344±0.0002^c^
C3	74.88±1.29^c^	0.0510±0,0005^e^	78.52±1.68^a^	0.0359±0.0002^c,d^
C4	75.43±1.32^c^	0.0423±0.0003^c^	79.35±0.49^a^	0.0345±0.0002^c^
C5	78.41±1.31^c^	0.0397±0.0002^f^	81.59±1.08^c^	0.0365±0.0008^a,d^
C6	78.42±0.42^c^	0.0393±0.0002^f^	81.83±1.45^c^	0.0320±0.0006^e^

GI_max_: Maximal inhibition of growth achieved after 24 h of treatment. GI_50_: concentration of the compound required to reduce the cell growth by 50% (relative to negative control). The results were obtained by the SRB assay and are shown as mean ± SEM of three independent experiments. Different letters in the same column indicate significant differences (*P *< 0.01) by Tukey’s test.

**Figure 2:**
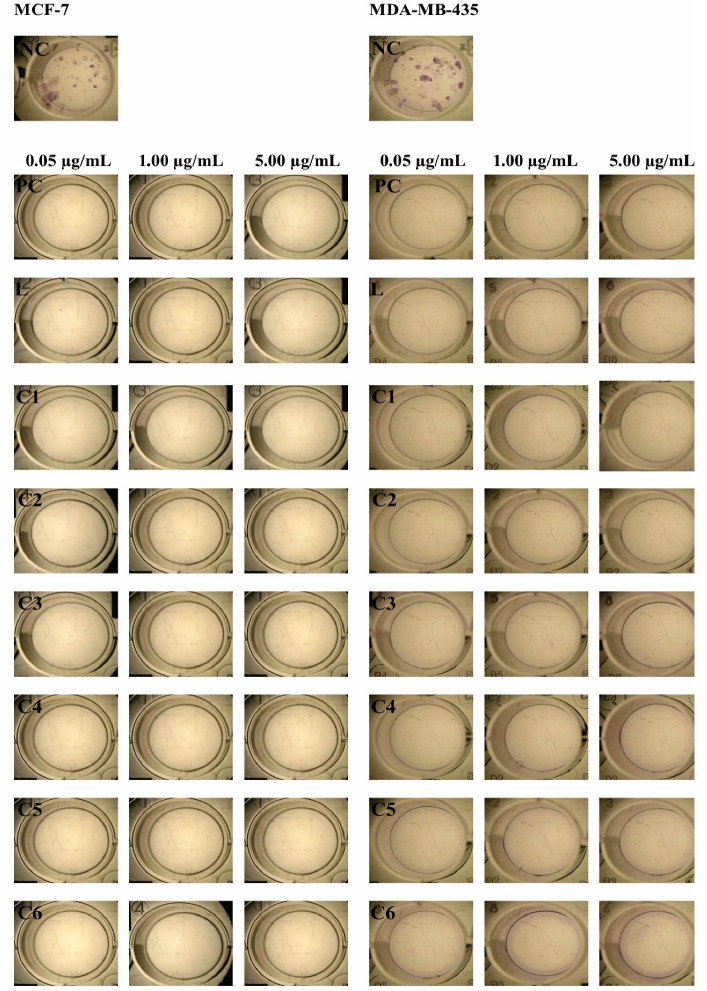
Inhibition of MCF-7 and MDA-MB-435 colony formation upon treatment with different compounds concentrations. NC, untreated; PC, cisplatin; L, ligand; C1-C6, Pd(II) complexes. The experiment was performed three times, and a representative experiment is shown.

 To test the long-term antiproliferative effect of Pd(II) complexes, we performed a clonogenic assay. After 14 days of culture, all compounds were able to interfere with the long-term proliferation and survival of both MCF-7 and MDA-MB-435 cells, completely inhibiting colony formation (Figure 2).

 Previous studies have also demonstrated the cytotoxic effect of palladium(II) imine ligand complexes, but, the complexes exhibited better activity under the experimental conditions tested herein against MCF-7 and MDA-MB-435 cells, compared to the other palladium complexes.^18-21^ All of the complexes tested exhibited antitumor activity, however, C5 and C6 had the lower IG50 which may be attributed, at least in part, to both the structure and substituent which is involved in the mechanism of ligand exchange in square-planar Pd and Pt compounds and it is very important for the stability of the Pd(II) complex in the biological media.^31^ Pd(II) complexes derived from Schiff base ligands with different chemical structures from ours also exhibited cytotoxic effects towards MCF-7^32-36^ and MDA-MB-435^37,38^ cells, highlighting the importance of Pd(II) substituent and donors for complexes activities.

 One interesting feature of our series of compounds was its capacity to reach maximum inhibition of growth values around 80%, against apoptosis-resistant MDA-MB-435 cells, demonstrated by the short-term growth inhibition analysis that was further confirmed by the clonogenic assay. This result indicated the potential antitumor effect of Pd(II) complexes, through inhibiting cell growth and consequently spreading.

###  Inhibition of cell migration and adhesion

**Figure 3:**
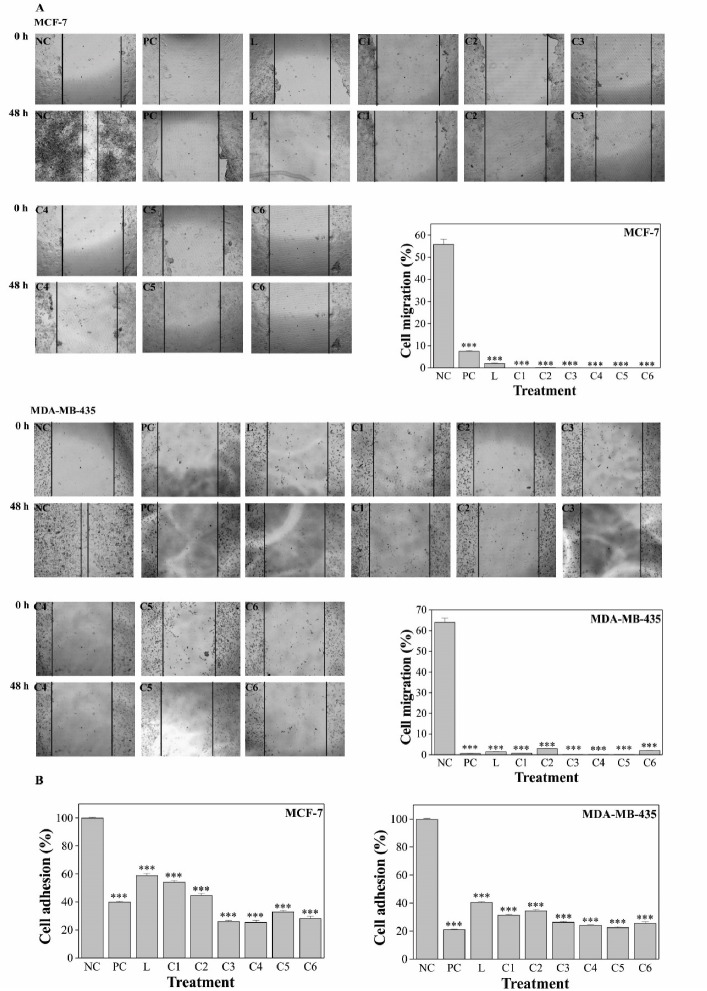
Pd(II) complexes inhibited MCF-7 and MDA-MB-435 cell migration (A) and adhesion (B). NC, untreated; PC, cisplatin; L, ligand; C1-C6, Pd(II) complexes. The compounds were used at 0.05 μg/mL. Results are expressed as mean ± SEM of three independent experiments. ***P< 0.001 versus the control group.

 The results indicated that Pd(II) complexes treatment inhibited MCF-7 and MDA-MB-435 cell migration significantly (*P* < 0.001) compared to the NC group. The complexes were more active against MCF-7 migration than the positive control drug cisplatin (PC) and the effect of complexes on MDA-MB-435 cells was equivalent to that of PC (Figure 3A). Cell adhesion was also significantly (*P* < 0.001) inhibited upon Pd(II) complexes treatment when compared to the NC group, for both cell lines MCF-7 and MDA-MB-435 (Figure 3B).

 Metastasis is one of the most harmful and difficult issues in cancer management and crucial progress of tumor cell metastasis relays on cell migration and adhesion. Adhesion is the first step in the process of tumor cell invasion, and the formation and disassembly of adhesion drive the migration cycle.^39,40^ The results revealed that the complexes could significantly suppress MCF-7 and MDA-MB-435 cells migration and adhesion which may indicate a positive role of Pd(II) complexes in the prevention of metastasis.

###  Morphological and morphometrics alterations and cell death

**Figure 4:**
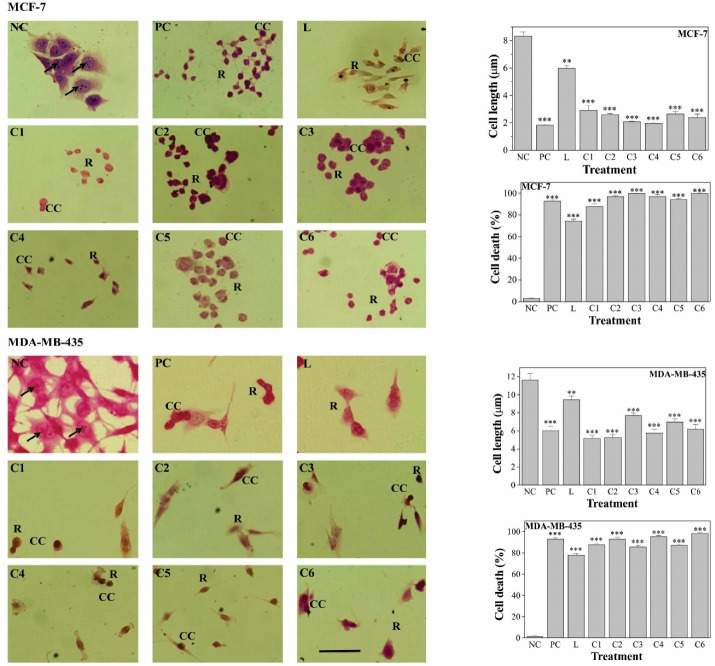
MCF-7 and MDA-MB-435 cell morphological and morphometric alterations and cell death induction upon treatment with 0.05 μg/mL Pd(II) complexes. NC, untreated; PC, cisplatin; L, ligand; C1-C6, Pd(II). Results are expressed as mean ± SEM of three independent experiments. **P<0.01; *** P<0.001 versus the control group. Scale bar: 10 μm.

 Pd(II) complexes treatment induced morphological alterations in both MCF-7 and, MDA-MB-435 cells, including cellular shrinkage, chromatin condensation, rounding-up and, also triggered cell death (Figure 4). MCF-7 negative control cells grow as colonies, presented irregular morphology, with the cytoplasm less stained than the nucleus and the nucleolus clearly visible. MDA-MB-435 negative control cells are irregular-shaped cells, with nuclear and, cytoplasmic pleomorphism, cytoplasm less stained than the nucleus and the nucleolus also clearly visible. After exposure to the compounds, cells exhibited morphological alterations that indicate cytoskeleton disruption. The Pd(II) complexes produced more conspicuous morphological changes than the ligand, for both cell lines. Cell shrinkage, an indicating feature of cell death, was additionally established by the determination of cell length, which was significantly (*P *< 0.01) decreased in comparison to control cells. Cell death induction upon treatment was further confirmed, by the Fast green-dye exclusion method, and the effect of Pd(II) complexes was equivalent to that of cisplatin.

 Cell morphology alterations, suggesting cell death induction, were also observed upon palladium(II) complexes, with Schiff bases derived from diverse ligands, treatment.^18,21,36,38^

###  DNA cleavage

**Figure 5:**
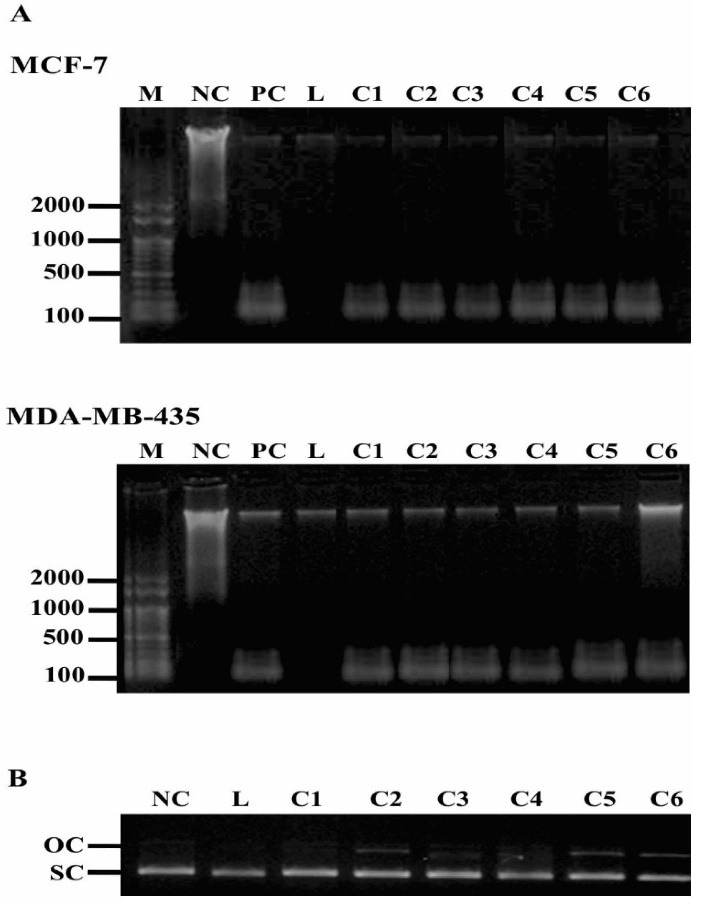
DNA cleavage activity of 0.05 μg/mL Pd(II) complexes. DNA laddering (A) indicating MCF-7 and MDA-MB-435 cells apoptosis and cleavage pattern of pUC19 plasmid DNA (B). M, DNA size marker (pb); NC: untreated; PC, cisplatin; L, ligand; C1-C6, Pd(II) complexes. The experiment was performed three times, and a representative experiment is shown.

 Pd(II) complexes treatment induced both cell DNA fragmentation and plasmid DNA cleavage (Figure 5). The fragmentation of genomic DNA indicated that both MCF-7 and, MDA-MB-435 cells had undergone apoptosis, as an internucleosomal cell DNA digestion occurred, a hallmark feature of apoptosis (Figure 5A). Pd(II) complexes also damaged circular double-stranded DNA, in a cell-free system. NC shows the SC form of plasmid DNA. On the addition of Pd(II) complexes, mainly C5 and C6, the OC form of plasmid DNA can be seen, indicating that some plasmid DNA cleavage occurred.

 DNA ladder pattern is used for the detection of apoptosis^41^ and the results indicated that the complexes indeed induced DNA fragmentation in both cell lines. Pd(II) complex with saccharinate also induced apoptosis in MCF-7^42^ and MDA-MB-435 cells, but in the last it was not observed DNA laddering formation.^38^ Moreover, our results showed that besides the high antiproliferative effect, the complexes also interact with DNA, as the degree of superhelicity of the plasmid DNA molecules has been altered and a significant cleavage of DNA takes place under the influence of complexes which might account for their cytotoxic effect against both MCF-7 and MDA-MB-435 cells. Similar alteration of the plasmid DNA tertiary structure was also observed with other Pd(II) complexes suggesting a similar mechanism of cisplatin action.^35,37^

 Disabling of apoptosis is a central event in tumorigenesis. Cell death resistance is responsible not only for tumor development and progression but also for tumor resistance to therapies that represent one of the major obstacles to successful cancer treatment. Therefore induction of cell death plays a central role in cancer treatment strategies.^43,44^

## Conclusion

 The results of the present work highlight the anticancer potential of the newly synthesized Pd(II) complexes, as they exhibited cytotoxic activities, including the ability to reduce cell viability, colony formation, migration and, adhesion of both MCF-7 and MDA-MB-435 cells, while also induced cell death. Therefore, Pd(II) complexes might be considered as potential candidates for antineoplastic drug development.

## Acknowledgments

 We thank FAPEMIG for financial support (Grants: CEX - APQ-01984-14 and Rede Mineira de Química- REDE-113/10) and the fellowship from FAPEMIG and CNPq (ISD), CAPES (EPG), CNPq (ETA) and from PET-MEC-SESu (CMCPG) were gratefully acknowledged.

## Author Contributions

**Conceptualization: ** Bruna Alexandre Oliveira da Silva, Isabela Spido Dias, Luís Eduardo Sarto, Elba Pereira de Gois, Claúdia Torres, Eduardo Tonon de Almeida, Cibele Marli Cação Paiva Gouvêa.

**Data curation: ** Cibele Marli Cação Paiva Gouvêa.

**Formal analysis: ** Bruna Alexandre Oliveira da Silva, Cibele Marli Cação Paiva Gouvêa.

**Funding acquisition: ** Eduardo Tonon de Almeida.

**Investigation:** Bruna Alexandre Oliveira da Silva, Isabela Spido Dias, Luís Eduardo Sarto, Elba Pereira de Gois, Claúdia Torres, Eduardo Tonon de Almeida, Cibele Marli Cação Paiva Gouvêa.

**Methodology: **Claúdia Torres, Eduardo Tonon de Almeida, Cibele Marli Cação Paiva Gouvêa.

**Project administration: ** Eduardo Tonon de Almeida, Cibele Marli Cação Paiva Gouvêa.

**Resources: **Eduardo Tonon de Almeida, Cibele Marli Cação Paiva Gouvêa.

**Supervision: **Eduardo Tonon de Almeida, Cibele Marli Cação Paiva Gouvêa.

**Validation: **Bruna Alexandre Oliveira da Silva, Isabela Spido Dias, Luís Eduardo Sarto, Elba Pereira de Gois, Claúdia Torres, Eduardo Tonon de Almeida, Cibele Marli Cação Paiva Gouvêa.

**Visualization: **Bruna Alexandre Oliveira da Silva, Isabela Spido Dias, Luís Eduardo Sarto, Elba Pereira de Gois, Claúdia Torres, Eduardo Tonon de Almeida, Cibele Marli Cação Paiva Gouvêa.

 Writing – original draft: Bruna Alexandre Oliveira da Silva, Cibele Marli Cação Paiva Gouvêa.

**Writing – review & editing: ** Bruna Alexandre Oliveira da Silva, Isabela Spido Dias, Luís Eduardo Sarto, Elba Pereira de Gois, Claúdia Torres, Eduardo Tonon de Almeida, Cibele Marli Cação Paiva Gouvêa.

## Ethical Issues

 Not applicable.

## Conflict of Interest

 The authors declare no conflicts of interest.
